# Attraction and Compaction of Migratory Breast Cancer Cells by Bone Matrix Proteins through Tumor-Osteocyte Interactions

**DOI:** 10.1038/s41598-018-23833-1

**Published:** 2018-04-03

**Authors:** Andy Chen, Luqi Wang, Shengzhi Liu, Yue Wang, Yunlong Liu, Mu Wang, Harikrishna Nakshatri, Bai-Yan Li, Hiroki Yokota

**Affiliations:** 1 Department of Biomedical Engineering, Indiana University Purdue University Indianapolis, Indianapolis, IN 46202 USA; 2 Department of Pharmacology, School of Pharmacy, Harbin Medical University, Harbin, 150081 China; 3 Department of Medical and Molecular Genetics, Indiana University School of Medicine, Indianapolis, IN USA; 4 Department of Biochemistry and Molecular Biology, Indiana University School of Medicine, Indianapolis, IN USA; 5 Department of Surgery, Simon Cancer Research Center, Indiana University School of Medicine, Indianapolis, IN 46202 USA

## Abstract

Bone is a frequent site of metastasis from breast cancer. To understand the potential role of osteocytes in bone metastasis, we investigated tumor-osteocyte interactions using two cell lines derived from the MDA-MB-231 breast cancer cells, primary breast cancer cells, and MLO-A5/MLO-Y4 osteocyte cells. When three-dimensional (3D) tumor spheroids were grown with osteocyte spheroids, tumor spheroids fused with osteocyte spheroids and shrank. This size reduction was also observed when tumor spheroids were exposed to conditioned medium isolated from osteocyte cells. Mass spectrometry-based analysis predicted that several bone matrix proteins (e.g., collagen, biglycan) in conditioned medium could be responsible for tumor shrinkage. The osteocyte-driven shrinkage was mimicked by type I collagen, the most abundant organic component in bone, but not by hydroxyapatite, a major inorganic component in bone. RNA and protein expression analysis revealed that tumor-osteocyte interactions downregulated Snail, a transcription factor involved in epithelial-to-mesenchymal transition (EMT). An agarose bead assay showed that bone matrix proteins act as a tumor attractant. Collectively, the study herein demonstrates that osteocytes attract and compact migratory breast cancer cells through bone matrix proteins, suppress tumor migration, by Snail downregulation, and promote subsequent metastatic colonization.

## Introduction

Bone is the most frequently metastasized site by breast cancer^1^. The bone microenvironment is rich in growth factors, such as insulin-like growth factor 1 (IGF1) and bone morphogenetic proteins (BMPs), as well as cytokines such as IL6, IL8 and IL11^2^. Tumor cells may initiate bone resorption and induce a “vicious cycle”, in which various growth factors are released from bone matrix to promote further bone resorption^3^. In the vicious cycle, transforming growth factor beta (TGFβ), abundant in the bone matrix and secreted by macrophages, plays a pivotal role in tumor-bone interactions^4^. TGFβ stimulates production of parathyroid hormone-related protein (PTHrP) in tumor cells, which elevates expression of the receptor activator of nuclear factor kappa B (RANKL) in bone-forming osteoblasts and activates bone-resorbing osteoclasts^5^. While preventing the vicious cycle in the bone microenvironment is essential for protecting bone from metastatic destruction, it is also important to evaluate the role of osteocytes, the most abundant cells in bone matrix.

Osteocytes are bone cells differentiated from bone-forming osteoblasts, and they make up over 90% of the cells in mineralized bone^6^. They are mechano-sensors, and in response to physical stimulation they reduce the synthesis of sclerostin, an inhibitor of bone formation^7,8^. To our knowledge, the role of osteocytes in the progression and metastasis of tumors is not fully understood. In this study, we employed two breast cancer cell lines, TMD and BMD tumor cells, which are clones of MDA-MB-231 breast cancer cells. TMD cells were isolated from the mammary tumor resulting from the injection of MDA-MB-231 cells to the mammary fat pad of NOD/SCHID mouse, while BMD cells were harvested from the metastasized bone^9^. Compared to BMD cells, it is reported that TMD cells exhibits higher cellular motility^10^.

In this study, we evaluated tumor-bone interactions by employing three types of bone cell lines: MC3T3 osteoblast-like cells^11^, MLO-A5 and MLO-Y4 osteocyte-like cells^12^, and RAW264.7 pre-osteoclast cells^13^. To evaluate physiologically relevant interactions, we mostly focused on interactions of three-dimensional (3D) BMD and TMD tumor spheroids with bone spheroids or conditioned media isolated from bone cell cultures^14^. We also used 3D bioprinting^15^ and examined migratory behaviors of BMD and TMD cells towards MLO-A5 spheroids. The temporal changes of tumor spheroids were monitored using IncuCyte ZOOM, a real-time, live-cell imaging system^16^.

The primary question we addressed in this study was: What morphological and expression changes do tumor-bone interactions induce in 3D tumor spheroids? Among the three types of bone cells, we mainly focused on tumor-osteocyte interactions, since both MLO-A5 and MLO-Y4 osteocyte-like cells significantly induced compaction of tumor spheroids. To understand the mechanism of compaction, we employed mass spectrometry and predicted potential secretory factors that are responsible for compaction in conditioned medium from MLO-A5 and MLO-Y4 cells. Bone matrix proteins biglycan^17^, osteonectin^18^, and type I collagen^19^ were identified as potential factors for compacting tumor spheroids. We investigated the regulation of bone matrix proteins using RNA sequencing and Western blot analysis and examined possible links to epithelial-to-mesenchymal transition (EMT) and regulation of Snail, a transcription factor involved in EMT^20^. We employed an agarose bead assay and evaluated the chemotactic attraction capability of bone matrix proteins to tumor cells^21^.

## Results

### Alterations of size and surface roughness of tumor spheroids by bone components

**Figure 1:**
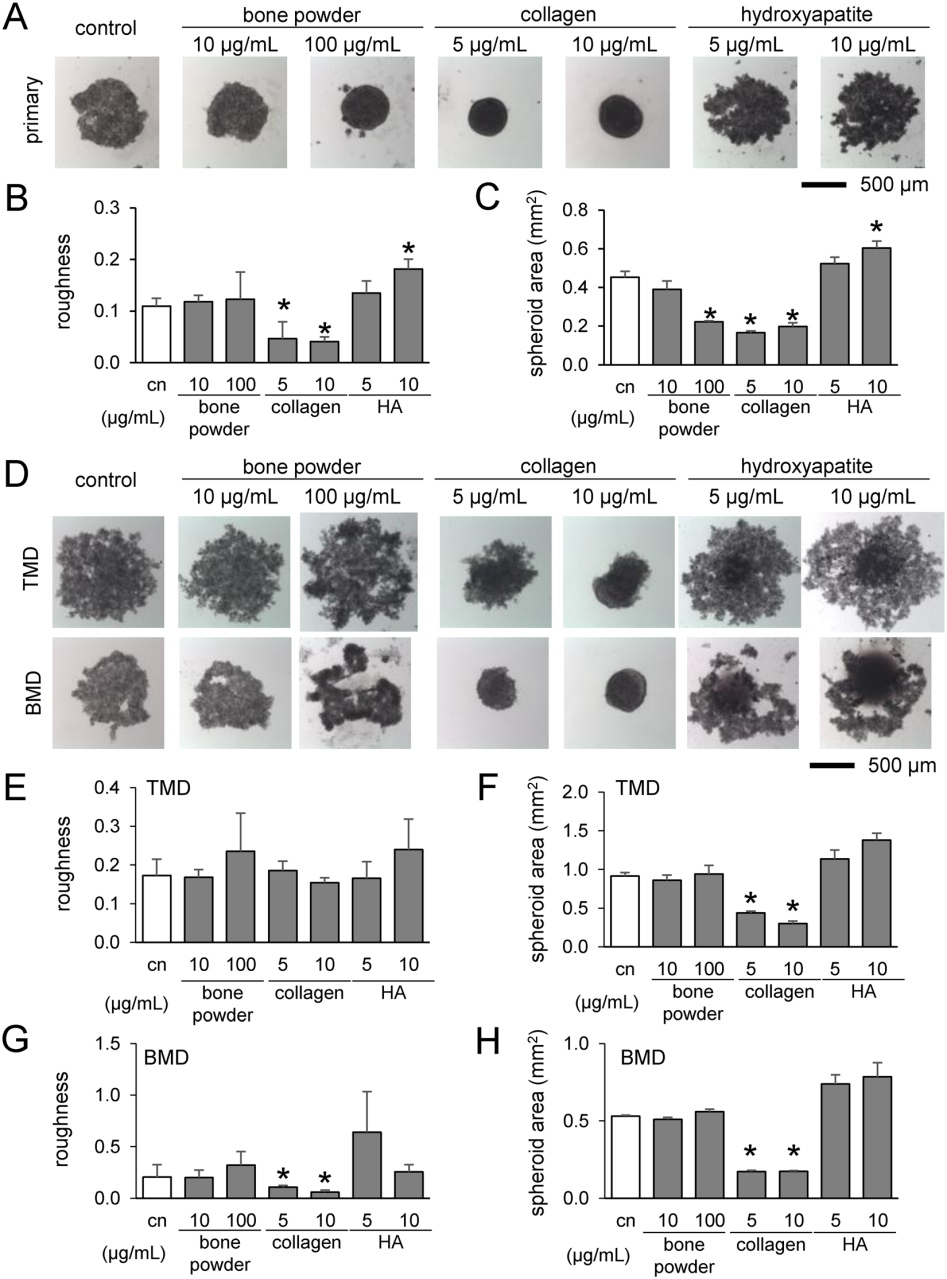
Formation of tumor spheroids in the presence of powdered bone extract (10 and 100 µg/mL), collagen (5 and 10 µg/mL), and hydroxyapatite (5 and 10 µg/mL) after 24 h. An asterisk (*) denotes *p* < 0.05 compared with control. (**A**) Tumor spheroids with primary breast cancer cells. (**B** & **C**) Roughness and cross-sectional area of primary cell spheroids, respectively. (**D**) Tumor spheroids with TMD and BMD cells. (**E** & **F**) Roughness and area of TMD cell spheroids. (**G** & **H**) Roughness and cross-sectional area of BMD cell spheroids.

Using primary breast cancer cells and TMD/BMD cell lines, we evaluated the effects of powdered bone extract (10 and 100 µg/mL), type I collagen (5 and 10 µg/mL), and hydroxyapatite (5 and 10 µg/mL) on formation of tumor spheroids. Of note, bone extracts contain both organic and inorganic components of mineralized bone, while type I collagen and hydroxyapatite are the major organic and inorganic components, respectively, in bone. With bone powder and collagen, primary breast cancer cells formed smaller spheroids, but hydroxyapatite caused spheroids to be larger (Fig. 1A–C). TMD and BMD cell spheroids responded similarly to collagen and hydroxyapatite, but bone powder had no significant effect (Fig. 1D–H). For all tumor cells, collagen reduced the roughness of spheroid surfaces.

### Compaction of tumor spheroids by osteoblast/osteocyte spheroids

**Figure 2:**
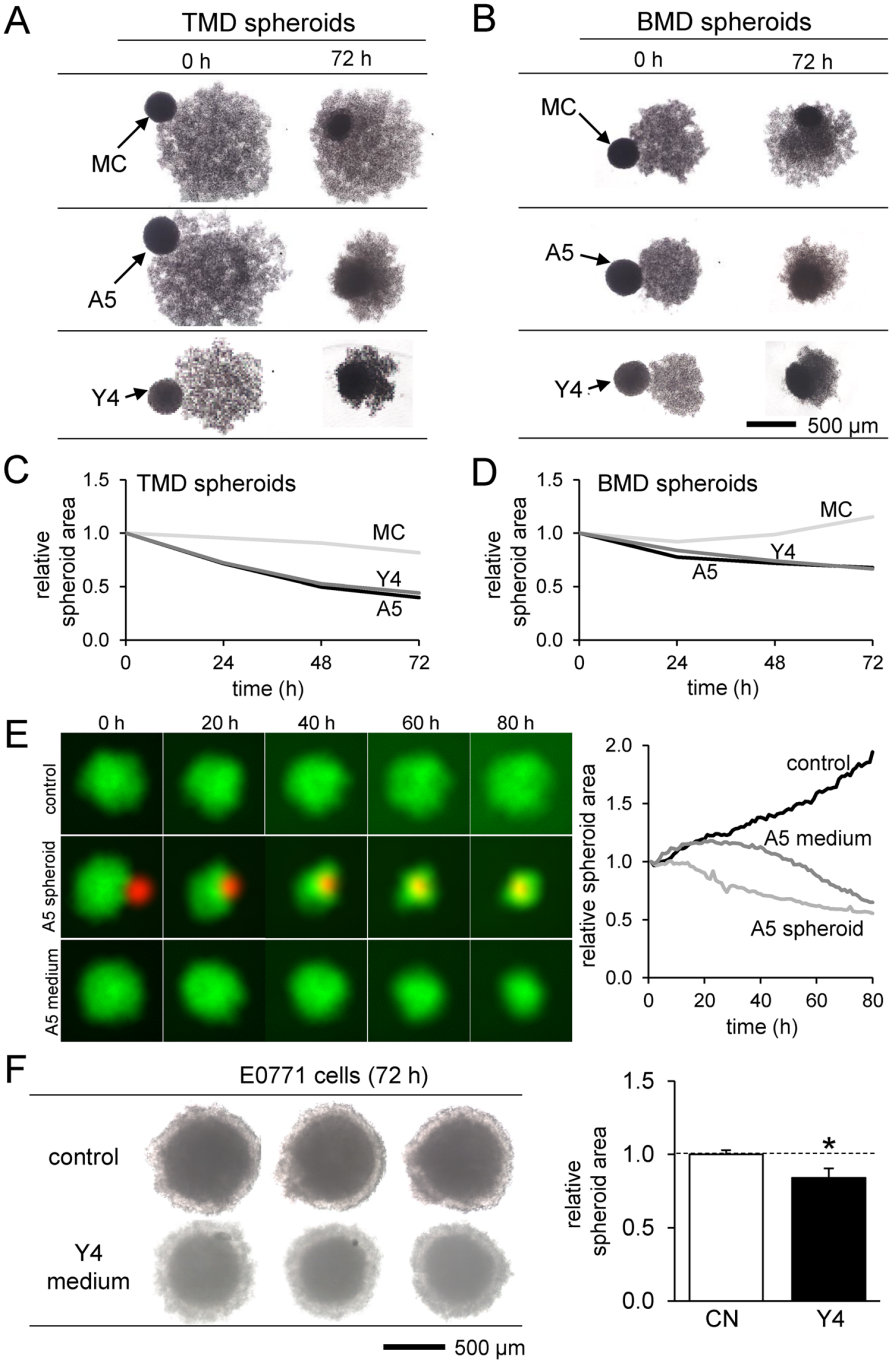
Temporal changes in the co-cultured tumor and bone spheroids. Of note, MC = MC3T3 osteoblast-like cells, and A5 = MLO-A5 osteocyte-like cells. (**A** & **B**) Shape changes by spheroid fusion of TMD or BMD tumor spheroids with MC3T3, MLO-A5, or MLO-Y4 bone spheroids. (**C** & **D**) Changes in the cross-sectional area of TMD and BMD spheroids, respectively. (**E**) Changes in the cross-sectional area of TMD spheroids (green). Control = no treatment, A5 fusion = fusion with MLO-A5 spheroid (red), and A5 media = culture in conditioned medium isolated from MLO-A5 osteocyte-like cells. (**F**) Shrinkage of E0771 spheroids by MLO-Y4 conditioned medium.

We next focused on osteoblasts and osteocytes and examined their direct and indirect effects on tumor spheroids. When TMD and BMD spheroids were cultured together with MC3T3, MLO-A5, or MLO-Y4 spheroids, the tumor spheroids engulfed the smaller bone spheroid and reduced their cross-sectional areas (Fig. 2A,B). In both cell lines, the tumor spheroids were compacted more by MLO-A5 and MLO-Y4 osteocyte-like spheroids than MC3T3 pre-osteoblast spheroids (Fig. 2C,D). Compaction of tumor spheroids was observed by direct interactions with MLO-A5 spheroids as well as indirect interactions via MLO-A5-derived conditioned medium (Fig. 2E). The degree of compaction was greater by direct interactions than indirect interactions. Besides TMD and BMD spheroids, E0771 mammary tumor spheroids also reduced their cross-sectional area in response to MLO-Y4-derived conditioned medium (Fig. 2F).

### Compaction of tumor spheroids by osteocyte-derived conditioned media

**Figure 3:**
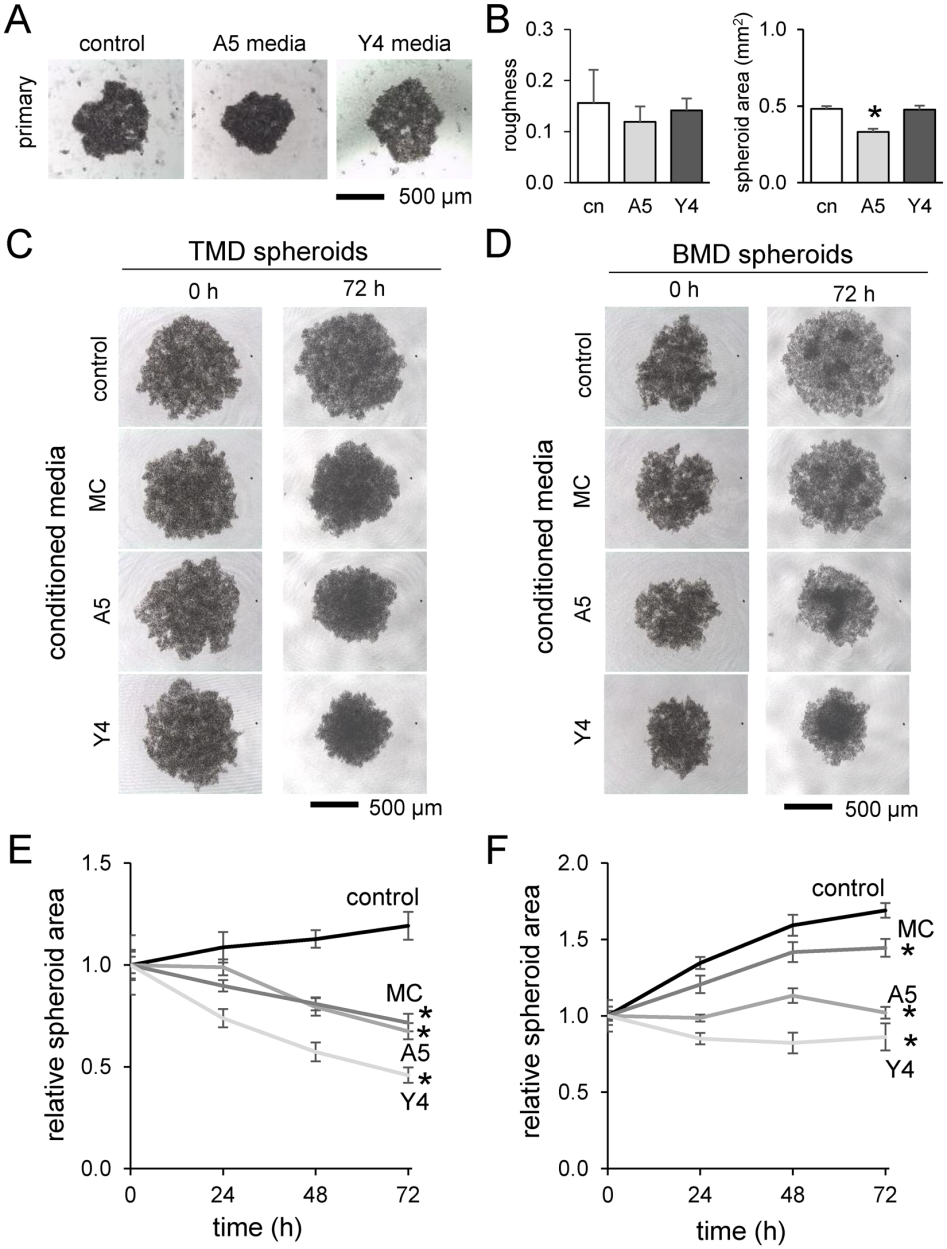
Effects of the conditioned medium on TMD and BMD spheroids. Of note, MC = MC3T3 osteoblast-like cells, A5 = MLO-A5 osteocyte-like cells, and Y4 = MLO-Y4 osteocyte-like cells. The asterisk indicates statistical significance at *p* < 0.05. (**A**) Primary cell spheroids formed in media conditioned with MLO-A5 and MLO-Y4 spheroids after 24 h. (**B**) Roughness and area of the primary cell spheroids. (**C** & **D**) TMD and BMD spheroids in the conditioned media isolated from MC3T3, MLO-A5, and MLO-Y4 cultures. (**E** & **F**) Relative size change of TMD and BMD spheroids in the conditioned media.

To further evaluate the indirect effect of osteoblasts and osteocytes on tumor spheroids, we employed conditioned medium isolated from MC3T3, MLO-A5, and MLO-Y4 cell cultures. We observed tumor cell-dependent responses. First, primary breast cancer cell spheroid formation was smaller in the presence of MLO-A5 conditioned media, but not MLO-Y4 conditioned media (Fig. 3A). Second, TMD tumor spheroids were significantly compacted by conditioned media from all three (*p* < 0.01) (Fig. 3C). Lastly, BMD tumor spheroids were also made smaller by the conditioned media (*p* < 0.01) (Fig. 3D). In both TMD and BMD spheroids, the osteocyte-derived conditioned media (MLO-A5 and MLO-Y4) tended to compact the tumor spheroids more than osteoblast-derived conditioned medium (MC3T3) (Fig. 3E,F).

### Tumor cell-dependent responses in 3D microenvironment

**Figure 4:**
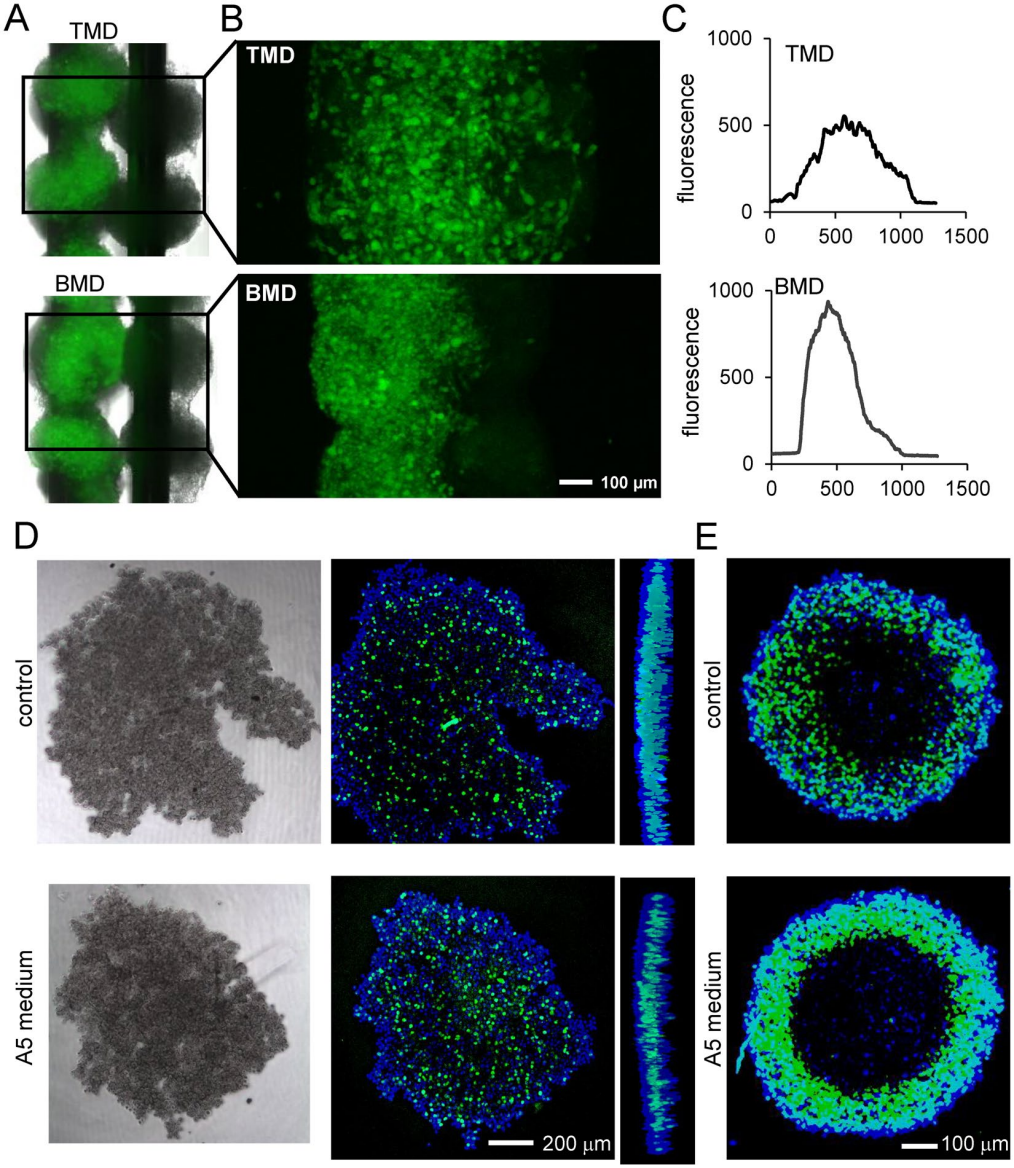
Three-dimensional characterization of tumor-osteocyte interactions. (**A** & **B**) TMD and BMD cell spheroids (green), positioned adjacent to MLO-A5 spheroids (black) 48 h after 3D bioprinting. (**C**) Average fluorescence intensity along the axis perpendicular to the supporting needle shaft. (**D** & **E**) Click-iT EdU assay of proliferating cells in 4T1.2 cell spheroids and TMD cell spheroids, respectively, in the presence and absence of MLO-A5-conditioned media.

To evaluate differential responses of TMD and BMD cells in the bone microenvironment, we generated a tumor-osteocyte hybrid construct using 3D bio-printing (Fig. 4A). The construct consisted of fluorescently-labeled TMD or BMD spheroids on one needle and MLO-A5 spheroids on the neighboring needle. After 48 h, the construct was imaged using confocal microscopy. Profiling the average fluorescence along the construct showed that TMD cells spread onto neighboring MLO-A5 spheroids more quickly than BMD cells (Fig. 4B,C).

Another comparison was conducted between TMD cells and 4T1.2 mammary tumor cells, which is known to spontaneously metastasize to bone in a mouse model. A Click-iT EdU cell proliferation assay was conducted on tumor spheroids in the presence of MLO-A5 conditioned media. In TMD cell spheroids, there was no significant difference in the pattern of green fluorescence-labeled DNA amplification in the cross-section of control and conditioned medium treated samples (Fig. 4D). In 4T1.2 cell spheroids, however, the conditioned media increased the number of proliferating cells, indicating that interactions of tumor cells with osteocytes stimulate proliferation of tumor cells in the bone microenvironment (Fig. 4E).

### Effects of EDTA on the compaction of TMD tumor spheroids

**Figure 5:**
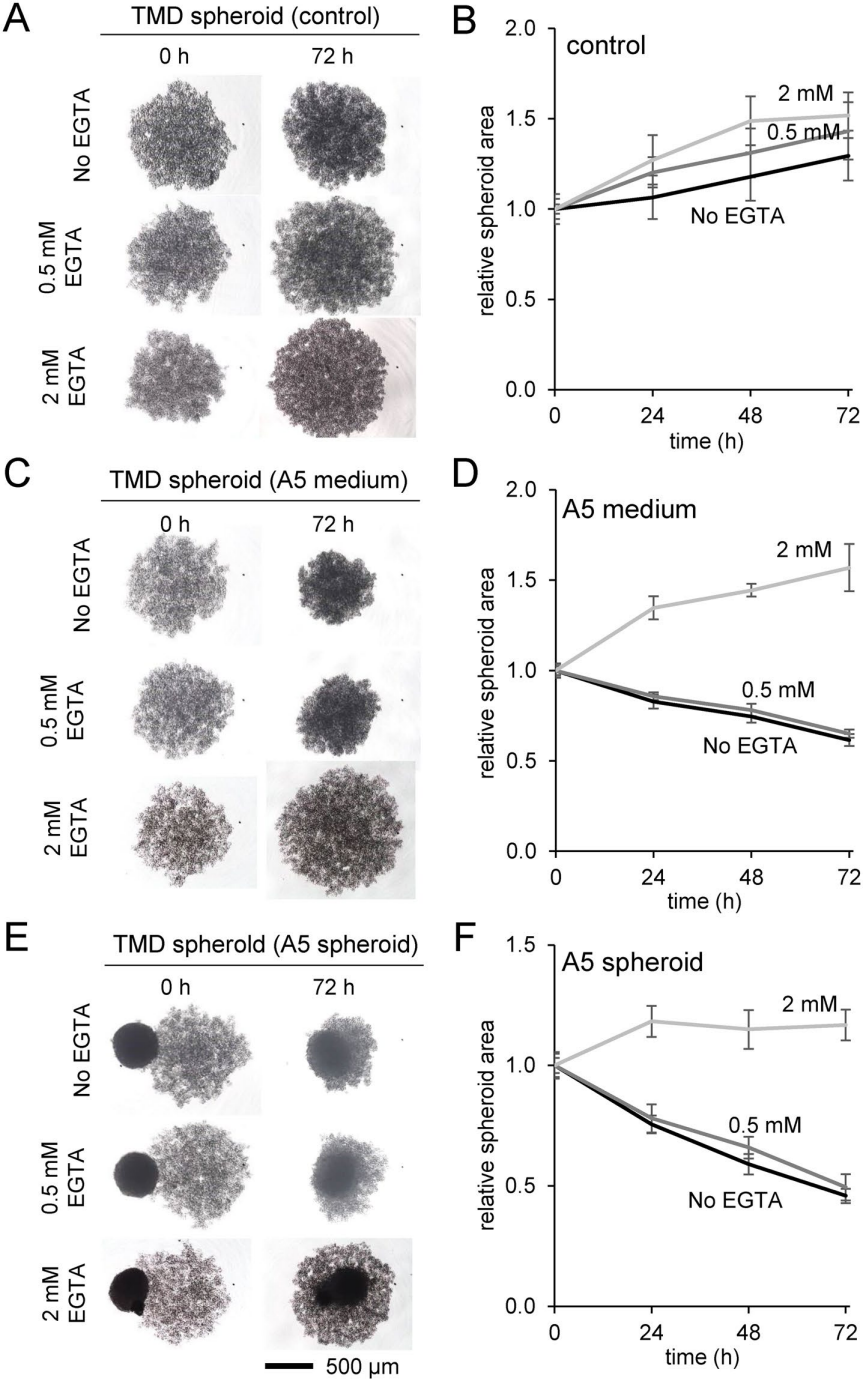
Effects of 0.5 and 2 mM EGTA on TMD tumor-osteocyte interactions. Of note, A5 = MLO-A5 osteocyte-like cells. (**A** & **B**) Size change of the control spheroids in 72 h. (**C** & **D**) Size change in response to the conditioned medium from MLO-A5 cell culture in 72 h. (**E** & **F**) Size change in response to MLO-A5 spheroid in 72 h.

Focusing on interactions of TMD tumor spheroids with MLO-A5 cells, we further evaluated calcium dependence of the spheroid compaction response in the presence of 0.5 and 2 mM EDTA. Consistent with the result in Fig. 3, the control spheroids showed no compaction (Fig. 5A,B), while compaction was induced by MLO-A5 conditioned medium (Fig. 5C,D) and fusion with MLO-A5 spheroids (Fig. 5E,F). Incubation with 0.5 mM EDTA did not show a detectable difference with control, but 2 mM EDTA significantly reduced the compaction by MLO-A5 cells.

### Predicting proteins in conditioned media responsible for tumor compaction

**Figure 6:**
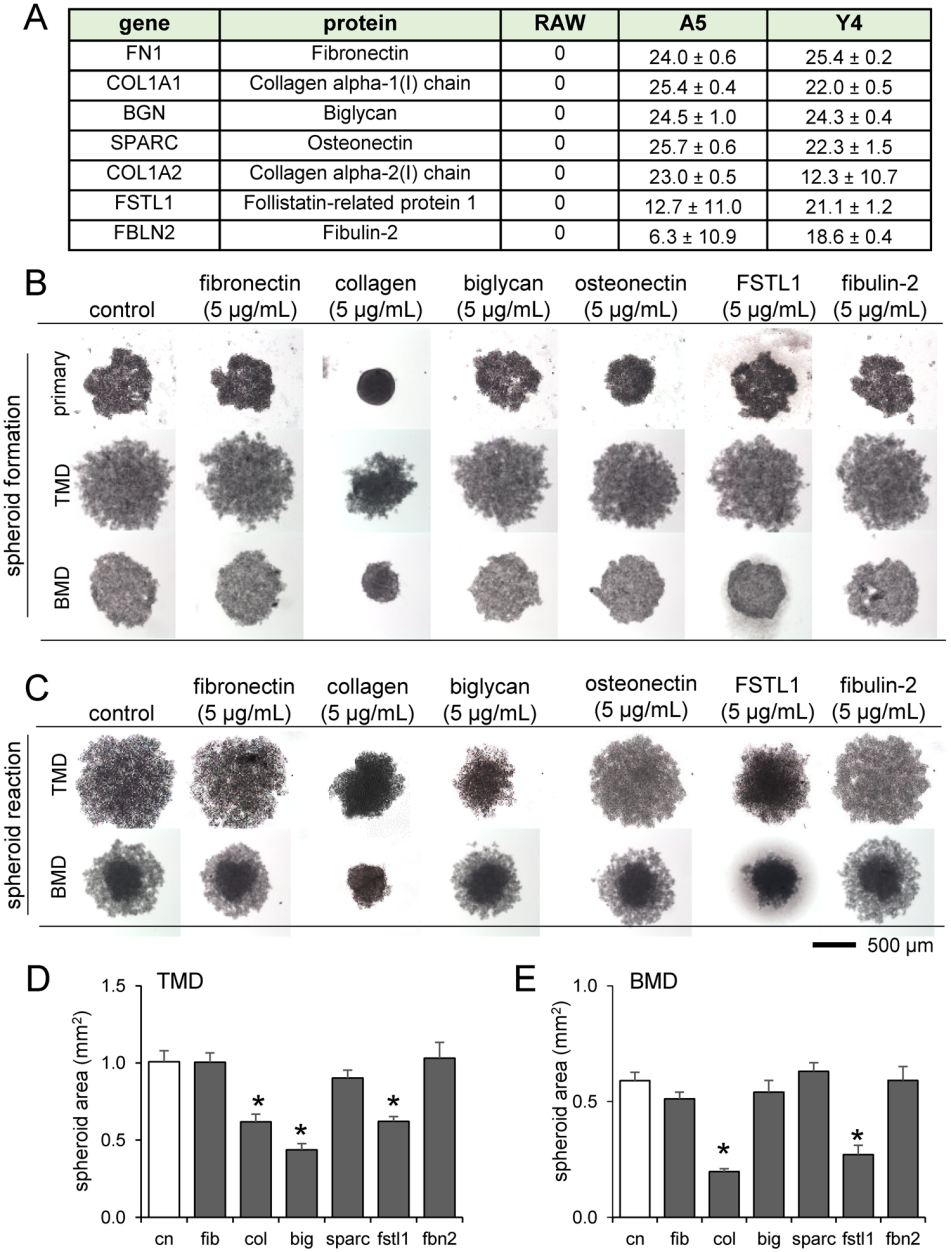
Effect of mass spectrometry-predicted proteins present in osteocyte-conditioned media on tumor spheroid formation. Of note, cn = control, fib = fibronectin, col = type I collagen, big = biglycan, SPARC = osteonectin, FSTL1 = follistatin-related protein 1, and fbn2 = fibulin 2. (**A**) List of proteins present in MLO-A5 and MLO-Y4 media but not in RAW264.7 media. (**B**) Primary, TMD, and BMD cell spheroid formation after 24 h in the presence of mass spectrometry-predicted proteins. (**C**) TMD spheroids 72 h after incubation in the presence of mass spectrometry-predicted proteins. (**D** & **E**) Changes in spheroid cross-sectional area in response to the mass spectrometry-predicted proteins. An asterisk (*) denotes statistical significance (*p* < 0.05) compared to control.

To identify the secretory factors in MLO-A5/Y4 conditioned media that compact tumor spheroids, we conducted protein analysis of three conditioned media (RAW264.7 as control, MLO-A5 and MLO-Y4) using mass spectrometry. The differential screening in the conditioned media predicted 7 bone matrix proteins as candidate dwarfing factors (Fig. 6A). Based on the prediction, we conducted formation of tumor spheroids in the presence of fibronectin, type I collagen, biglycan, osteonectin, follistatin-related protein 1 (FSTL1), or fibulin 2 (Fig. 6B). Fibronectin, osteonectin, and fibulin 2 are glycoproteins that bind ECM components such as collagen^18,22,23^. Biglycan is a leucine-rich repeat proteoglycan and rich in bone^17^, while FSTL1 binds TGFβ and is involved in skeletal development^24^. Spheroid reaction was also measured by adding each of these proteins to the spheroid media after 48 h of formation (Fig. 6C). The results demonstrated that for all kinds of tumor spheroids (primary, TMD, and BMD cells), collagen, biglycan, and osteonectin decreased spheroid size similarly to osteocyte-conditioned media, though the degree of decrease varied among the three kinds of tumor cells (Fig. 6D,E).

### Linkage to EMT-related genes

**Figure 7:**
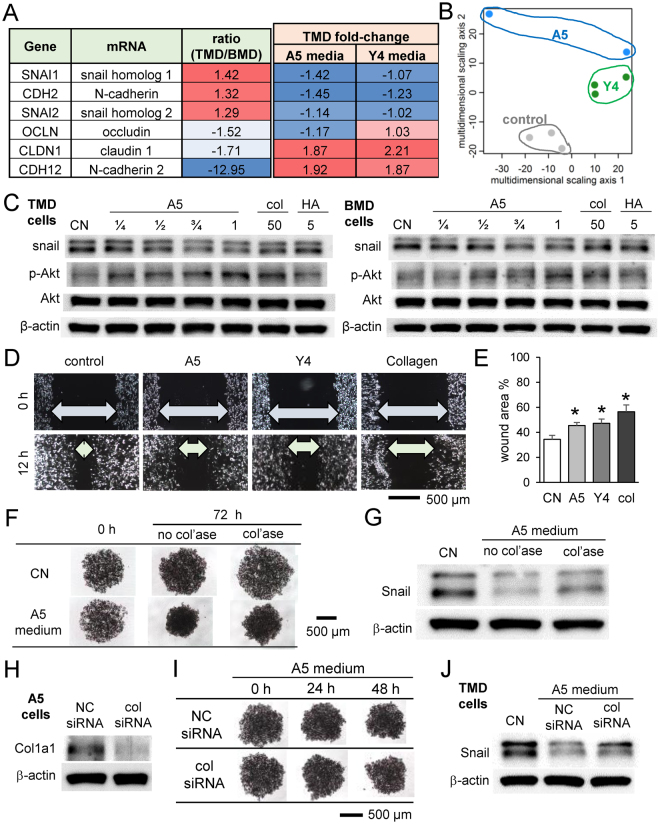
Effect of MLO-A5 conditioned medium, collagen, and hydroxyapatite on expression of Snail and p-Akt. (**A**) Comparison of mRNA levels of EMT genes in TMD and BMD tumor cells, and the TMD response to MLO-A5 and MLO-Y4 conditioned media. (**B**) Multi-dimensional scaling analysis of TMD response to MLO-A5 and MLO-Y4 conditioned media. (**C**) Expression changes of Snail and p-Akt in response to MLO-A5 conditioned media, collagen, and hydroxyapatite administration. Full-length Western blot images are presented in Supplementary Fig. S3. (**D** & **E**) Effects of collagen administration on the wound healing of TMD cells. (**F**) Effect of collagenase-treated conditioned medium on TMD tumor spheroids. (**G**) Effect of collagenase-treated conditioned medium on Snail expression. (**H–I**) Effect of col1a1 siRNA on TMD tumor spheroids and Snail expression.

The basal gene expression of TMD and BMD cells was assessed by cDNA microarray, and TMD response to MLO-A5 and MLO-Y4 conditioned media was evaluated by RNA sequencing (Fig. 7A). Snail, N-cadherin, and Slug mRNA, three EMT-linked genes, were more highly expressed in TMD cells and decreased by osteocyte-conditioned media. Claudin 1 and Occludin, two tight junction-related genes, were more highly expressed in BMD cells, but only claudin 1 was upregulated by both osteocyte conditioned media in TMD cells. Multi-dimensional scaling analysis of TMD response to MLO-A5 and MLO-Y4 conditioned media demonstrated differential response by group along the second MDS axis, but not the first MDS axis (Fig. 7B), suggesting that the effect of osteocyte-conditioned media on gene expression is an important driver of the differences among the cells. Western blot analysis of protein expression revealed that Snail expression was decreased by MLO-A5 conditioned media and collagen, but not by hydroxyapatite administration, in both TMD and BMD cells (Fig. 7C). Furthermore, phosphorylated Akt (p-Akt) was upregulated by MLO-A5 conditioned media as well as collagen, but not by hydroxyapatite (Fig. 7C). Administration of collagen as well as MLO-A5 and MLO-Y4-derived conditioned media suppressed the rate of wound healing of TMD cells in a scratch assay that measures cell migration (Fig. 7D,E).

We next inhibited collagen function in MLO-A5-derived conditioned medium by collagenase treatment and examined the response in tumor-osteocyte interactions. The result showed that collagenase-treated conditioned medium partially suppressed shrinkage of tumor spheroids (Fig. 7F). Furthermore, downregulation of Snail by MLO-A5-derived conditioned medium was partially restored by collagenase treatment (Fig. 7G). We also treated MLO-A5 osteocytes with siRNA specific to type I collagen α1 and examined the effect of siRNA-treated conditioned medium (Fig. 7H). Consistent with the collagenase treatment, silencing of type I collagen α1 partially suppressed osteocyte-driven downregulation of Snail expression (Fig. 7I,J). Of note, the cross-sectional area of tumor spheroids was not significantly different between non-specific control siRNA and col1α1 siRNA. Collectively, the results support the notion that collagen is involved in the response of tumor spheroids to osteocyte-derived conditioned media, and snail attenuation by MLO-A5 osteocytes is in part induced by collagen.

### Chemotactic migration of TMD cells

**Figure 8:**
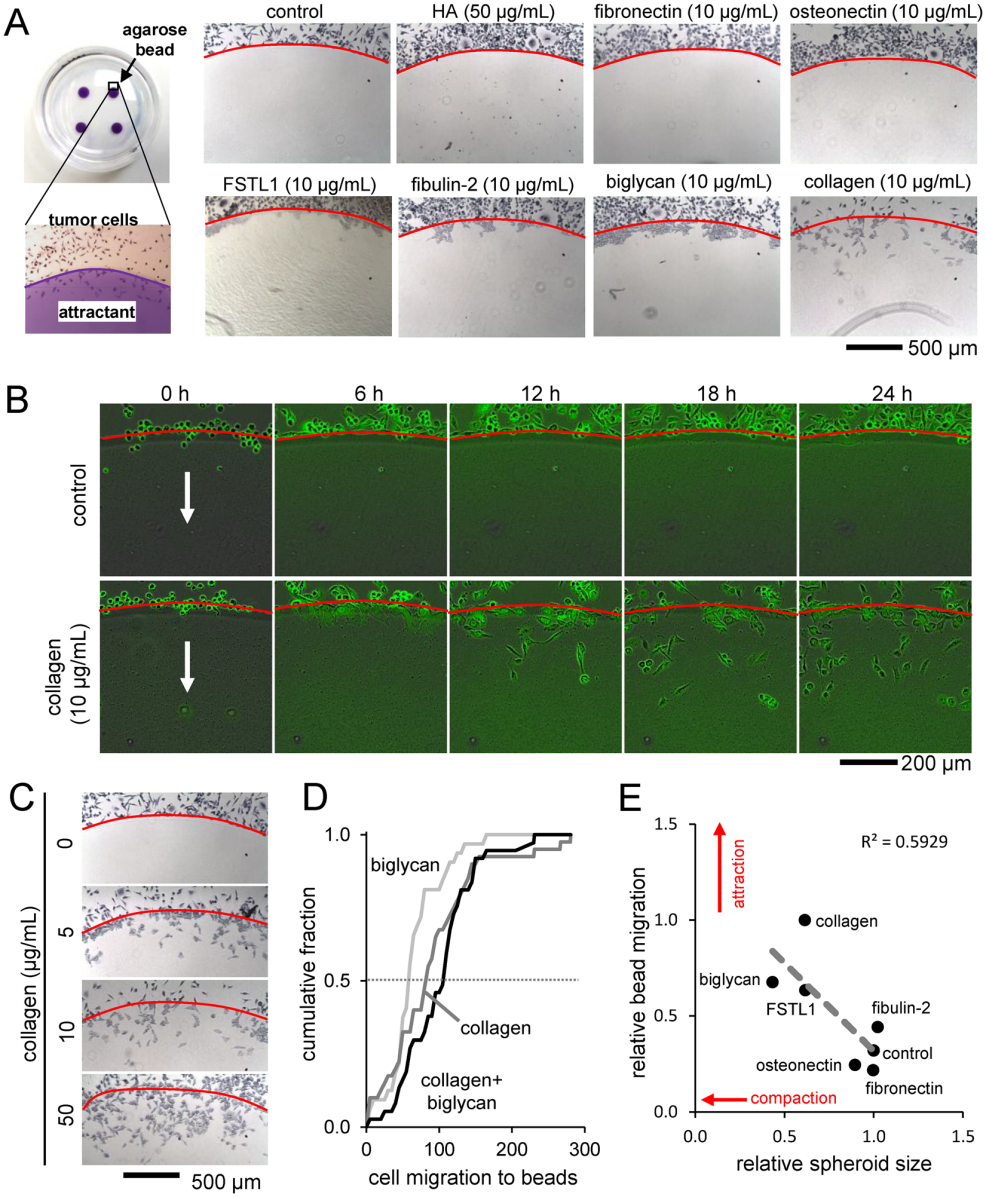
Agarose bead assay. (**A**) Images of the edge of the agarose beads showing migration of TMD tumor cells under the beads loaded with various bone matrix proteins and compounds. (**B**) Time response of TMD cells migrating under control and collagen-loaded agarose beads. (**C**) Images of the edge of agarose beads in response to increasing concentrations of collagen. (**D**) Cumulative fraction of cell migration at the agarose bead edge for collagen, biglycan, and collagen biglycan combination. (**E**) Relative spheroid size correlates with relative bead migration by treatment with bone matrix proteins.

To further investigate the role of collagen in tumor-osteocyte interaction, we conducted an agarose bead assay in which chemotactic migration of TMD cells was evaluated using an agarose hemisphere consisting of bone microenvironment-related compounds. TMD spheroids co-cultured with beads containing collagen, biglycan, fibulin 2, and FSTL1 tended to migrate under the bead, but did not with control beads and those with hydroxyapatite, osteonectin, and fibronectin (Fig. 8A). Using the Incucyte ZOOM real-time imaging system, the time course of TMD cell migration under control and collagen-loaded agarose beads was found over 24 h (Fig. 8B). TMD migration under the agarose bead increased in a dose-dependent manner as the collagen concentration in the bead increased (Fig. 8C). The combination of biglycan and collagen in the agarose bead increased the median number of migrating TMD cells compared to beads with only collagen (82 vs 103), though this difference was not statistically significant (Fig. 8D). The relative TMD spheroid size against relative migration under the bead after treatment with various bone proteins were found to be correlated (R^2^ = 0.59), where more spheroid shrinkage indicated more migrating cells (Fig. 8E). To demonstrate the release of collagen from the agarose bead, the diffusion coefficient was evaluated (Supplementary Fig. S2).

## Discussion

This study demonstrates that osteocytes act as an attractant as well as a spheroid compacting agent to MDA-MB-231-derived breast cancer cell lines. In the 3D spheroid assay, tumor spheroids significantly shrank in the presence of co-cultured osteocyte spheroids or MLO-A5 osteocyte-derived conditioned media. Mass spectrometry analysis identified several bone matrix proteins in MLO-A5-conditioned media as potential secretory factors responsible for tumor compaction, including collagen, biglycan, and osteonectin. Treatment with collagen and biglycan mimicked the tumor spheroid compaction induced by osteocyte spheroids or osteocyte-derived conditioned medium. Direct and indirect interactions with osteocytes reduced the expression of Snail, a transcription factor involved in EMT, raising the hypothesis that tumor interactions with osteocytes induce a reversal of EMT in the bone microenvironment.

Among the three osteoblast/osteocyte cell lines MC3T3 (pre-osteoblast), MLO-A5 (post-osteoblast, pre-osteocyte), and MLO-Y4 (mature osteocyte), we observed that conditioned media from more differentiated cells tended to shrink tumor spheroids more extensively. Contrary to the compaction effect of collagen, hydroxyapatite, a major inorganic component in mineralized bone, slightly expanded tumor spheroids. The compaction was observed with BMD and TMD tumor cells, as well as human primary breast cancer cells. TMD cells were isolated from tumors of injected MDA-MB-231 in a mouse, while BMD cells were isolated from bone metastasis after cardiac injection of TMD cells^9^. Our previous work showed that TMD cells exhibited more migratory characteristics *in vitro* than BMD cells^10^. Of note, primary human breast cancer cells are estrogen receptor-positive (ER+)^25^, while TMD and BMD cells are ER-negative. Thus, osteocyte-driven tumor compaction does not seem to be specific to the activity of estrogen receptor.

Mass spectrometry analysis followed by 3D spheroid-based validation revealed that collagen and biglycan are two candidate proteins responsible for compacting tumor spheroids. Collagen is the most abundant protein in bone, while biglycan is a leucine-rich repeat proteoglycan consisting of chondroitin sulfate and dermatan sulfate^17^. It interacts with collagen and binds to TGFβ, and its knockout in mice is reported to lead to an osteoporosis-like phenotype^26^. Biglycan is reported to induce inflammatory reactions and stimulate tumor cell migration via nuclear factor κB and extracellular signal-regulated kinase 1/2^27^. In our 3D spheroid assay, the degree of compaction differed between TMD and BMD cells, and it is possible that shrinkage responses are dependent on varying types of tumor cells. It is also possible that these matrix proteins contribute additively or synergistically to compacting tumor spheroids, and protein modifications such as glycosylation and phosphorylation may play important roles^28^.

We observed that migratory TMD tumor cells expressed a higher level of Snail than non-migratory BMD tumor cells, and its expression was downregulated by MLO-A5 conditioned medium as well as incubation with collagen. Snail is a zinc finger transcription factor that regulates cellular adhesion and promotes EMT^20^. The result herein indicates the potential linkage of the observed tumor compaction to the downregulation of Snail. Since the observed increase in p-Akt may stimulate proliferation of tumor cells, osteocytes and collagen may contribute to attracting tumor cells and allowing for proliferation in the bone microenvironment, while hydroxyapatite in calcified bone matrix may act as a repellent of tumor cells in bone.

Bone metastasis occurs after a series of events that allows the primary tumor cells to colonize the secondary site. The presented work examines some of the interrelated processes that are involved in this outcome. After a migratory tumor cell is transported to the bone, it undergoes actions that allow it to invade and thrive at the metastatic site. Spheroid formation and wound healing migration assays demonstrated that osteocytes may release factors that promote organization and slow migration. The agarose bead assay demonstrated that bone protein biglycan, like collagen, can act as chemoattractants for tumor cells. We found that collagen, biglycan, fibulin 2, and FSTL1 can induce tumor cell invasion under the bead, though only collagen seemed to allow cells to migrate through the bead. The other proteins allowed cells to penetrate the bead border, but the cells tended to aggregate there. Changes in spheroid shrinkage and bead migration by protein treatment was found to be correlated (Fig. 8E), demonstrating that the mechanisms behind these two phenomena may be related. It is possible that integrins such as α_1_β_1_, α_2_β_1_, and α_11_β_1_ may mediate interactions of type I collagen with tumor cells^29^, and other bone matrix proteins may assist their interactions through crosslinking collagens^30^. Further work can be done to investigate how tumor cells react differently to collagen and the other bone proteins.

This work demonstrates the involvement of osteocyte-secreted bone matrix protein in the bone microenvironment to interact with tumor cells. Though this is only one piece of the puzzle, this knowledge may lead to further work in characterizing how the bone microenvironment interacts with invading tumor cells. While the presented result reveals a novel feature of tumor-osteocyte interactions, the study has a few limitations. Our experiments used mouse bone cells and human cancer cells, and the potential effects of cross-species interactions should be taken into consideration. Furthermore, the effect of protein modification needs to be analyzed in tumor-bone matrix interactions. For example, recombinant biglycan from Chinese hamster ovary cells was used in this study, but its conjugation with chondroitin sulfate or dermatan sulfate might be evaluated to further understand biglycan’s action on tumor cells.

In summary, this study demonstrated that osteocyte-conditioned media compacted tumor spheroids and decreased EMT-related protein expression. Collagen was identified by mass spectrometry in the media and presented both tumor-compacting ability and Snail expression inhibition. Taken together, this work shows that osteocytes interact with tumor cells and alter adhesive and migratory behaviors of 3D tumor spheroids. The result on tumor-osteocyte signaling might contribute to developing novel therapies to prevent bone metastasis associated with breast cancer.

## Materials and Methods

### Cell culture

MDA-MB-231 human breast cancer cell-derived cell lines, TMD cells, BMD cells, and E0771 mammary tumor cells (CH3 BioSystems, Amherst, NY, USA) were grown in DMEM (Corning, Inc., Corning, NY, USA), and MC3T3 osteoblast-like cells, MLO-A5 and MLO-Y4 osteocyte-like cells were grown in αMEM (Gibco, Carlsbad, CA, USA). For TMD, BMD, and MC3T3 cells, the culture media was completed with 10% fetal bovine serum (FBS) and antibiotics (50 units/ml penicillin, and 50 µg/ml streptomycin; Life Technologies, Carlsbad, CA, USA). For MLO-A5 cells, the culture media contained 5% FBS and 5% fetal calf serum (FCS) with antibiotics; for MLO-Y4 cells, the culture media contained 2.5% FBS and 2.5% FCS with antibiotics. Primary breast cancer cells were prepared as previously described and cultured in a 3:1 v/v mixture of F-12 and DMEM supplemented with 5% FBS, 0.4 µg/mL hydrocortisone, 5 µg/mL insulin, 8.4 ng/mL cholera toxin, 10 ng/mL epidermal growth factor, 24 µg/mL adenine, and 5 µM Y-27632^25,31^. Cells were maintained at 37 °C and 5% CO_2_ in a humidified incubator. To measure the gene and protein expression levels in 2D, cells were seeded on 6-cm tissue culture dishes (Corning). After 48 h, cells were harvested.

### Knockdown of Col1α1 by siRNA

MLO-A5 osteocyte cells were treated with siRNA specific to Col1α1 (Cat No. 4390771, Life Technologies). A negative siRNA (Silencer Select #1, Life Technologies) was used as a nonspecific control. Cells were transiently transfected with siRNA using Lipofectamine RNAiMAX (Life Technologies) in Opti-MEM I medium.

### Spheroid formation, fusion, reaction, and conditioned media exchange assays

To induce spheroid formation, cells were cultured in a U-bottom low-adhesion 96-well plate (S-Bio, Hudson, NH, USA). All spheroid assays were performed in complete αMEM (10% FBS, 1% antibiotics). To observe spheroid fusion, spheroids were formed in separate wells for 48 h, then one spheroid was carefully transferred to the other’s well. To observe the effects of conditioned media, spheroids were formed in separate wells for 48 h, and the media was removed and replaced with conditioned media. To observe the spheroid reaction to chemical treatment, after 48 h of spheroid formation, media was removed and fresh media with the relevant chemicals were added. Conditioned media were treated with EGTA (Cat No. S311, Thermo Fisher Scientific, Waltham, MA, USA), type I collagen (Cat No. 344236, BD Biosciences, San Jose, CA, USA), or collagenase (Cat No. C9891, Sigma, St. Louis, MO, USA). Every 24 h, microscope images were taken of the spheroids to be analyzed with ImageJ. A threshold was applied and the spheroid area was identified with the “Analyze Particle” function. Area and circularity of the identified object were measured directly with ImageJ. Roughness was calculated by fitting an ellipse to the spheroid and adding the areas of the spheroid outside the ellipse and the areas of the ellipse not within the spheroid.

### Bioprinted constructs

Spheroids of 5 × 10^3^ TMD and BMD cells (fluorescent stained with Incucyte CytoLight Green, Essen Bioscience, Michigan, USA) co-cultured with 1.2 × 10^4^ MLO-A5 cells along with spheroids of solely 1.2 × 10^4^ MLO-A5 cells were formed for 48 h. The spheroids were bioprinted onto a needle array with a Regenova 3D Bioprinter (Cyfuse Biomedical, Tokyo, Japan) such that co-cultured tumor/bone spheroids were placed adjacent to bone-only spheroids. Fluorescence microscope images were taken every 24 h, and confocal microscope images were acquired at 48 h with FV1000 (Olympus, Tokyo, Japan). Images were analyzed with ImageJ.

### Mass spectrometry protein identification

Three dishes of each of RAW264.7 cells, MLO-A5 cells, and MLO-Y4 cells (~1 × 10^6^ cells each) were cultured for 2 days, and their conditioned media were harvested and freeze-dried. To predict secretory factors for compacting tumor spheroids, proteins in the freeze-dried samples were analyzed by reverse-phase HPLC-ESI-MS/MS using the Dionex-Thermo Fisher Scientific UltiMate 3000 RSLC nano System (Thermo Fisher Scientific) coupled to the Q-Exactive HF Hybrid Quadrupole Orbitrap MS (Thermo Fisher Scientific)^32^.

### cDNA microarray and RNA sequencing

Using cDNA microarrays (Human Gene 2.0 ST, Affymetrix), genome-wide mRNA expression profiles were determined using RNA isolated with an RNeasy Plus Mini kit (Qiagen, Germantown, MD, USA) from 9 samples, including 3 samples each from 3 groups of cells (MDA-MB-231 parental cells, TMD cells, and BMD cells). RNA sequencing was performed on RNA from TMD cells incubated with control, MLO-A5-conditioned media, and MLO-Y4 conditioned media. After cDNA library construction using TruSeq Stranded mRNA Library Prep kit (Illumina, San Diego, CA, USA), the NextSeq500 (Illumina) sequenced the samples. Quality control was performed using FastQC (Babraham Bioinformatics, Cambridge, UK), and the sequenced libraries were mapped to the UCSC hg19 human genome. Multidimensional scaling (MDS) was performed on the log2-transformed expression data.

### Western blot analysis

Cells were lysed in a radio-immunoprecipitation assay (RIPA) buffer. Isolated proteins were fractionated using 10% SDS gels and electro-transferred to polyvinylidene difluoride membranes (Millipore, Billerica, MA, USA). We used antibodies against Snail (Cell Signaling), and β-actin (Sigma). Protein levels were assayed using a SuperSignal west femto maximum sensitivity substrate (Thermo Fisher Scientific).

### Agarose bead assay

Agarose beads were formed by pipetting 10 µL warmed liquid 2% agarose onto 3.5 cm cell culture dishes. After adding 2 × 10^5^ tumor cells to each dish, they were incubated in 37 °C cell incubator for 24 h. Cells were fixed by 5 min incubation with 70% ethyl alcohol then by 5 min incubation with Giemsa stain (Sigma). Images were taken at the edges of the beads, and the numbers of cells inside the border of the agarose bead were counted.

### Statistical analysis

Three or four independent experiments were conducted and data were expressed as mean ± S.D. Statistical significance was evaluated using one-way analysis of variance (ANOVA). Post hoc statistical comparisons with control groups were performed using Bonferroni correction with statistical significance set at *p* < 0.05. The single and double asterisks in the figures indicate *p* < 0.05 and *p* < 0.01, respectively.

The datasets generated during and/or analyzed during the current study are available from the corresponding author on reasonable request.

## Electronic supplementary material

Supplementary Information
