# Supplementary material for: Attraction and Compaction of Migratory Breast Cancer Cells by Bone Matrix Proteins through Tumor-Osteocyte Interactions
Source: Sci Rep. 2018 Apr 3;8:5420. doi: 10.1038/s41598-018-23833-1 (PMC5882940; doi:10.1038/s41598-018-23833-1)
Supplement: Supplementary file 1 — Supplementary Information [file 41598_2018_23833_MOESM1_ESM.docx]

**Supplementary Information**

**Attraction and Compaction of Migratory Breast Cancer Cells**

**by Bone Matrix Proteins through Tumor-Osteocyte Interactions**

Andy Chen^1^, Luqi Wang^1, 2^, Shengzhi Liu^1, 2^, Yue Wang^1, 2^, Yunlong Liu^3^, Mu Wang^4^,

Harikrishna Nakshatri^5^, Bai-Yan Li^2^, and Hiroki Yokota^1, 2^

^1^Department of Biomedical Engineering, Indiana University Purdue University Indianapolis, Indianapolis, IN 46202, USA

^2^Department of Pharmacology, School of Pharmacy, Harbin Medical University, Harbin 150081, China

^3^Department of Medical and Molecular Genetics, Indiana University School of Medicine, Indianapolis, IN, USA

^4^Department of Biochemistry and Molecular Biology, Indiana University School of Medicine, Indianapolis, IN, USA

^5^Department of Surgery, Simon Cancer Research Center, Indiana University School of Medicine, Indianapolis, IN 46202, USA

| 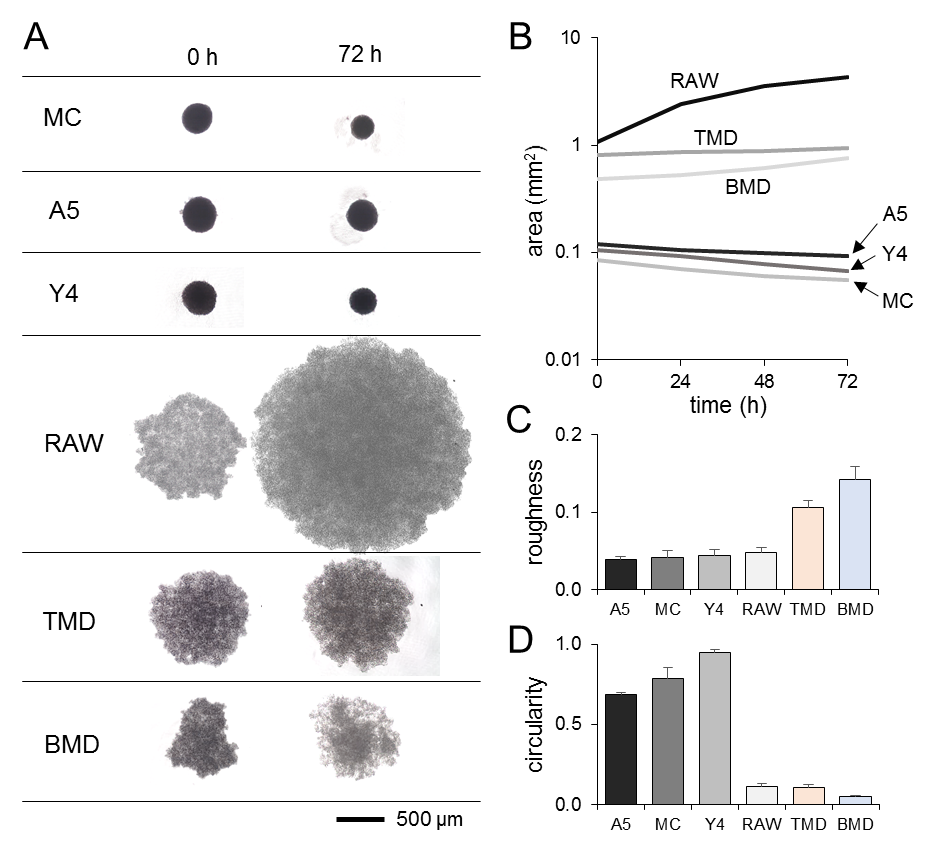 |
| --- |
| **Supplementary Figure S1**. Characterization of spheroids with bone cells and tumor cells. (A) Spheroid formation at 0 h and 72 h. Of note, MC = MC3T3 osteoblast-like cells, A5 = MLO-A5 osteocyte-like cells, Y4 = MLO-Y4 osteocyte-like cells, RAW = RAW264.7 preosteoclast cells, and TMD and BMD = Two clones of MDA-MB-231 breast cancer cells. (B) Changes in cross-sectional areas. (C) Roughness of spheroid surface. (D) Circularity of spheroids. |

| 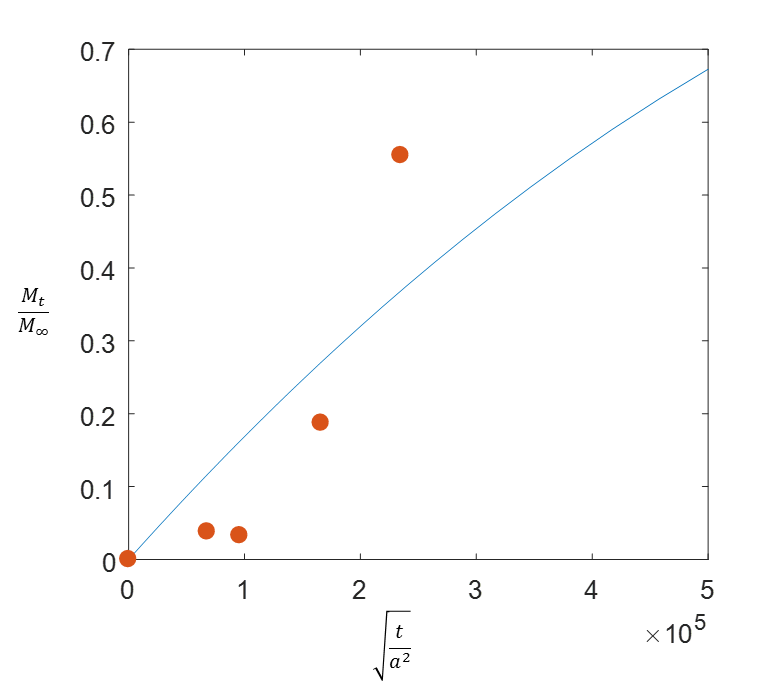 |
| --- |
| **Supplementary Figures S2**. Estimation of the diffusion coefficient of collagen in an agarose bead. Suppose the spherical agarose bead with a radius of $a$ (~0.17 cm) and an initial concentration of collagen at $C_{0}$ (1 mg/ml) is confined in a dish that initially contains no collagen. Collagen is transferred from the bead to the dish by diffusion and the concentration of collagen in the dish is always kept uniform. The total amount of transferred collagen $M_{t}$ after time $t$ is expressed (J. Crank. The Mathematics of Diffusion. Oxford Science Publications. 2^nd^ Edition, 1975):  $\frac{M_{t}}{M_{\infty}}=1-\sum_{n=1}^{\infty} \frac{6\alpha\left( 1+\alpha\right)exp\left( -\frac{Dq_{n}^{2}t}{a^{2}} \right)}{9+9\alpha+q_{n}^{2}\alpha^{2}}$ (Eq. S1)  in which $D$ = diffusion coefficient, and $M_{\infty}$ = total amount of collagen transfer at $t=\infty$. A set of parameters $q_{n}$ are non-zero roots of $tanq_{n}$, and $\alpha$ is defined:  $tanq_{n}=\frac{3q_{n}}{3+\alpha q_{n}^{2}}$ (Eq. S2) $\alpha=\frac{3M_{\infty}}{4\pi a^{3}C_{0}-3M_{\infty}}$ (Eq. S3)  In our experiment, $\alpha$ = 20. From the plot of $\frac{M_{t}}{M_{\infty}}$ vs. $\frac{\sqrt{t}}{a}$, the least root-mean-square estimate of $D$ is 2.5×10^-9^ cm^2^/sec. Alternatively, the diffusion coefficient can be estimated using Stokes-Einstein equation and a mean frictional drag coefficient, $f$:  $D=\frac{k_{B}T}{f}$ (Eq. S4) $f\cong\frac{4\pi\mu L}{ln\frac{L}{r}}$ (Eq. S5)  where $k_{B}$ = Boltzmann constant (1.38×10^-23^ m^2^kg/[s^2^K]), $T$ = absolute temperature (310 K), $f$ = friction coefficient for a cylindrical rod. Assuming $\mu$ = dynamic viscosity (~0.02 Pa·s = 20 cps) (Ellis, *et. al.*, Food Hydrocolloids, 2017, 73:222-228), $L$ = length of collagen (300 nm), and $r$ = radius of collagen (1 nm), the estimated value of $D$ from Eqs. S4 & S5 is 3.3×10^-9^ cm^2^/s. |

| 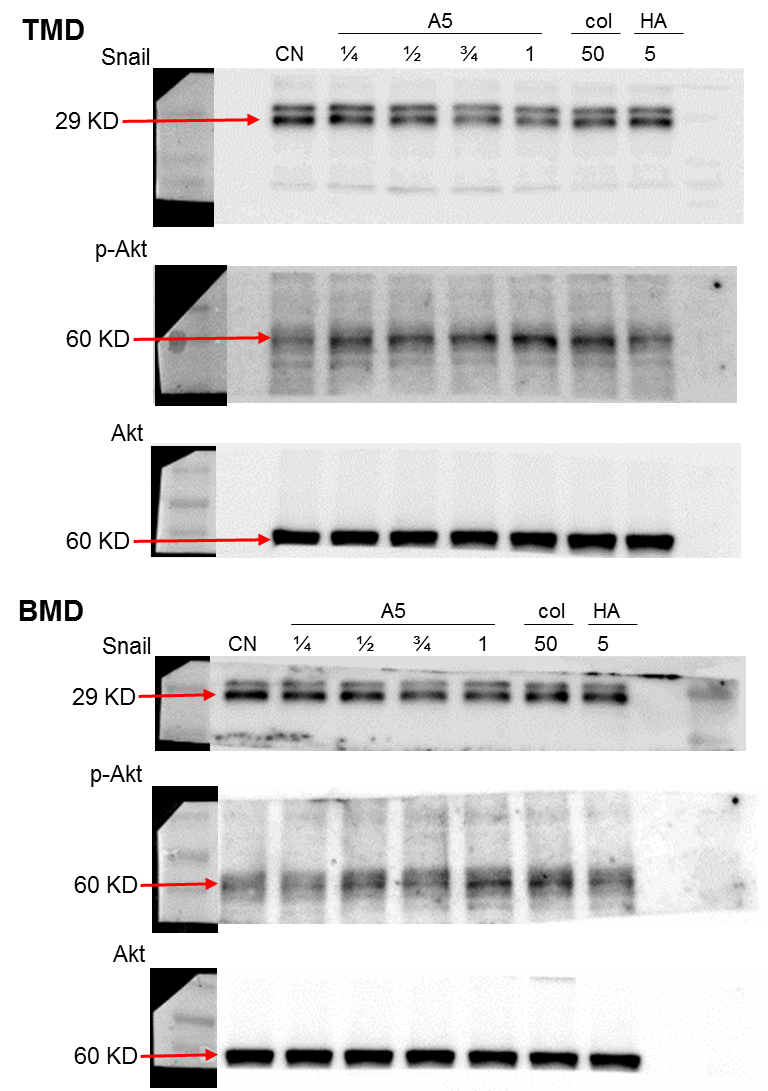 |
| --- |
| **Supplementary Figure S3.** Uncropped Western blot images for Figure 7C. |
